# Differential Visual Processing of Animal Images, with and without Conscious Awareness

**DOI:** 10.3389/fnhum.2016.00513

**Published:** 2016-10-13

**Authors:** Weina Zhu, Jan Drewes, Nicholas A. Peatfield, David Melcher

**Affiliations:** ^1^School of Information Science, Yunnan UniversityKunming, China; ^2^Department of Psychology, Giessen UniversityGiessen, Germany; ^3^Center for Mind/Brain Sciences (CIMeC), University of TrentoRovereto, Italy; ^4^Kunming Institute of Zoology, Chinese Academy of SciencesKunming, China; ^5^Department of Biomedical Physiology and Kinesiology, Simon Fraser UniversityBurnaby, BC, Canada

**Keywords:** continous flash suppression (CFS), natural scenes, ERPs (event-related potentials), awareness, animal detection

## Abstract

The human visual system can quickly and efficiently extract categorical information from a complex natural scene. The rapid detection of animals in a scene is one compelling example of this phenomenon, and it suggests the automatic processing of at least some types of categories with little or no attentional requirements (Li et al., 2002, 2005). The aim of this study is to investigate whether the remarkable capability to categorize complex natural scenes exist in the absence of awareness, based on recent reports that “invisible” stimuli, which do not reach conscious awareness, can still be processed by the human visual system (Pasley et al., 2004; Williams et al., 2004; Fang and He, 2005; Jiang et al., 2006, 2007; Kaunitz et al., 2011a). In two experiments, we recorded event-related potentials (ERPs) in response to animal and non-animal/vehicle stimuli in both aware and unaware conditions in a continuous flash suppression (CFS) paradigm. Our results indicate that even in the “unseen” condition, the brain responds differently to animal and non-animal/vehicle images, consistent with rapid activation of animal-selective feature detectors prior to, or outside of, suppression by the CFS mask.

## Introduction

The human visual system has a remarkable capability to extract categorical information from complex natural scenes, and the processing required for the recognition of objects and scenes seems to be instantaneous and effortless. When we view an image, we can usually understand what is displayed very quickly and easily. One particularly compelling example of this ability comes from animal categorization tasks in which participants respond whether or not there is an animal in the scene (Thorpe et al., 1996; Kirchner and Thorpe, 2006; Drewes et al., 2011; Zhu et al., 2013). In a go/no-go manual reaction task with briefly flashed images (20 ms), observers performed the task with approximately 92% accuracy and with reaction times as short as 390 ms (Thorpe et al., 1996). Saccadic latencies in a comparable two-alternative forced choice (2AFC) task were even shorter than the manual reaction times; subjects on average took only 228 ms to indicate which one of two images contained an animal. The shortest reaction times for which subjects could reliably identify the target image were on the order of 120 ms (Kirchner and Thorpe, 2006). This has given rise to the assumption that a feed-forward sweep through the ventral stream might be the neural mechanism responsible for this phenomenon (Thorpe et al., 1996; Kirchner and Thorpe, 2006; Crouzet and Serre, 2011).

This kind of rapid visual processing does not require foveal vision, and images can be presented at different eccentricities without a concurrent cost in accuracy (Fabre-Thorpe et al., 1998a; Thorpe et al., 2001; Drewes et al., 2011). For example, it has been reported that images presented in the far periphery at 70.5°, were correctly classified well above chance (60.5%; Thorpe et al., 2001). Furthermore, it has been shown that stimulus location uncertainty, lateralization and familiarity did not affect the speed of visual target processing (Fabre-Thorpe et al., 2001; Fize et al., 2005; Drewes et al., 2011). This ultra-rapid categorization has been found under various complex stimulus conditions, including objects embedded in natural scenes, such as animals, food, fish and trees (Fabre-Thorpe et al., 1998b; Vogels, 1999), and artificial objects such as various vehicles: cars, aircraft, boats, etc. (VanRullen and Thorpe, 2001b).

In addition to behavioral data showing remarkable speed and accuracy for categorization of novel natural scenes, evidence for ultra-rapid scene processing has been provided from event-related potentials (ERPs). Thorpe et al. (1996) found that the ERPs of animal and non-animal images show a distinct difference within 150 ms after stimulus onset. On the other hand, Johnson and Olshausen (2003) found a second component in the responses to a rapid scene processing task. Besides the early component (about 135 ms), they found a late component (150–300 ms) that more closely correlated with classification performance (Johnson and Olshausen, 2003).

All of the above findings were reported on visible, consciously perceived natural scenes. Recently, it has been shown that some stimuli that do not reach conscious awareness and are thus “invisible” are still processed to some degree by the visual system. Both the extraction of low-level visual features, such as orientation (Montaser-Kouhsari et al., 2004; Rajimehr, 2004; Bahrami et al., 2008), spatial information (van Boxtel et al., 2010), motion (Kaunitz et al., 2011a; for review see, Lin and He, 2009) and the binding of low-level visual features based on Gestalt grouping cues, such as good continuation and proximity (Mitroff and Scholl, 2005) can occur in the absence of awareness. It has also been reported that “high-level” stages of visual processing may be possible without being aware of the percept, for example in face inversion (Jiang et al., 2007; Zhou et al., 2010; Stein et al., 2011), face expressions (Jiang et al., 2009; Smith, 2012), semantic information (Jiang et al., 2007; Costello et al., 2009; Kang et al., 2011; but see Moors et al., 2016) and information integration (Mudrik et al., 2011, 2014). In fMRI studies, under interocular suppression, emotional faces generated stronger responses in the amygdala than neutral faces (Williams et al., 2004) and non-face objects (Pasley et al., 2004), and suppressed images of tools have been reported to activate the dorsal cortex (Fang and He, 2005; although the fMRI findings are controversial: Hesselmann and Malach, 2011). These findings suggest that considerable amounts of information, including at least some object category-related information, can be processed even when visual stimuli do not reach conscious awareness.

However, despite a host of research about ultra-fast processing in object recognition, few studies about rapid object detection in the absence of conscious awareness have been reported to date. One study of rapid categorization under continuous flash suppression (CFS) did not find evidence for category specific processing of unseen stimuli (Kaunitz et al., 2011b). In that experiment, participants viewed images of animals or tools, as well as scrambled images. In the case of visible images, both ERP and multivariate pattern (MVPA) analyses were able to distinguish between intact and scrambled images within around 100–125 ms. In contrast, neither the EEG or MVPA analyses could distinguish between intact and scrambled images in the unseen condition. However, it should be noted that in that study, visible and invisible stimuli had different levels of contrast. The authors of that study concluded that further work was needed to compare EEG signals when the stimulus was matched but only behavioral reports differed from trial to trial.

Another study reported that subjects can rapidly detect animals or vehicles in briefly presented novel natural scenes while simultaneously performing another attention-demanding task. They concluded that some visual tasks associated with “high-level” cortical areas, such as object detection, may proceed in the “near absence” of attention (Li et al., 2002, 2005). According to the unconscious unbinding hypothesis, some complex visual features with high behavioral relevance (such as fearful/angry faces or nudes) can be processed outside of awareness and draw greater attention or processing resources to the suppressed stimulus (Lin and He, 2009). Thus, we hypothesize that at least some aspects of the processing involved in rapid object detection might still be possible in the absence of awareness. In particular, categorization in terms of animate vs. non-animate targets is an interesting case in which to study visual processing outside of awareness. The animate/inanimate distinction has been described as fundamental based on neuropsychological evidence (Mahon et al., 2009) and measures of representational similarity in fMRI data (Kriegeskorte et al., 2008). Moreover, there is evidence for a selective categorization response for animal stimuli in the human amygdala (Mormann et al., 2011). These findings suggest that there are feature-detectors specifically tuned to animal images.

The aim of the present study was to investigate the mechanisms involved in rapid object recognition and to examine whether certain target categories, such as animals, are afforded privileged processing in the visual system. Repeatedly, animal stimuli have been shown to differ from non-animal stimuli in aspects like response time and the attraction of attentional resources (New et al., 2007; Ohman, 2007; Mormann et al., 2008; Mahon et al., 2009; Crouzet et al., 2012; Yang et al., 2012; Drewes et al., 2015), which may suggest a privileged kind of processing for animal stimuli. If these differences exist in relatively early stages of visual processing then they might also be found in the absence of awareness.

The 2AFC task is a classical paradigm in the investigation of ultra-rapid object categorization (Fabre-Thorpe et al., 1998a, 2001; VanRullen and Thorpe, 2001a; Johnson and Olshausen, 2003; Zhu et al., 2013). In the current study, we recorded ERPs during a 2AFC paradigm under a CFS. CFS is a variant of binocular rivalry in which a series of different Mondrian patterns are continuously flashed to one eye at a steady rate, causing a static low contrast image presented to the other eye to be reliably suppressed throughout the entire viewing period (Tsuchiya and Koch, 2005; Tsuchiya et al., 2006). Here CFS was used to reliably suppress natural scenes from conscious perception during the experiment. In contrast to the earlier study of animal categorization under CFS (Kaunitz et al., 2011b), target contrast was equated across seen and unseen trials. By recording ERPs during CFS, we investigated the mechanisms for the processing of visual stimuli in the presence or absence of conscious awareness.

## Ethics Statement

All experiments were approved by the local Ethics Committee of the Kunming Institute of Zoology, Chinese Academy of Sciences, and performed according to the principles expressed in the Declaration of Helsinki. Written informed consent was obtained from all participants.

## Experiment 1

In this experiment, we compared evoked responses to animal and non-animal stimuli in both seen and unseen conditions.

### Methods

#### Subjects

A group of 16 subjects participated in Experiment 1 (10 male, 6 female, aged 21–26: mean = 24.6, SD = 1.09). In all experiments, the participants were students or postdoctoral fellows recruited from Yunnan University or Kunming Institute of Zoology and were paid for their participation. All participants had normal or corrected-to-normal vision and were naive to the purpose of the experiment.

#### Apparatus

In all experiments, visual displays were presented on a 19″ ViewSonic CRT monitor (1024 × 768 pixels resolution, 100 Hz frame rate). Subjects viewed images through a mirror stereoscope, with their heads stabilized by a chin-and-head rest. The viewing distance was 57 cm. To achieve good fusion of the display, the mirrors were adjusted for each observer. Visual stimuli were presented in MATLAB (The MathWorks, Inc., 2012) using the Psychophysics Toolbox (Brainard, 1997; Pelli, 1997; Kleiner et al., 2007).

#### Stimuli and Procedures

**Figure 1:**
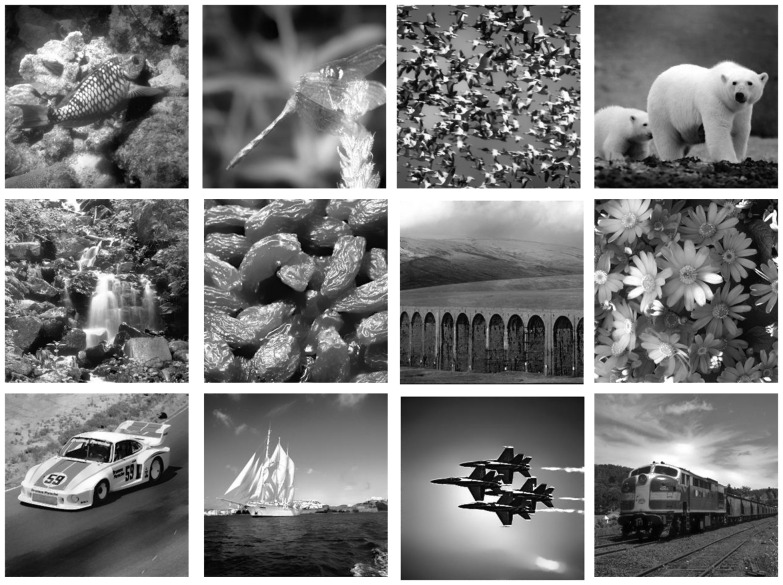
**Sample images of animal (top), non-animal (middle) and vehicle (bottom) stimuli**.

Three hundred animal images and 300 non-animal images were selected from the COREL stock photo library. The images in each category were chosen to be as varied as possible. The images were converted from RGB color to gray-scale using MATLAB’s built-in rgb2gray routine, which is a simple linear combination of the RGB color channels (0.2989 * R + 0.5870 * G + 0.1140 * B). We equated the luminance and contrast of the images by using the SHINE toolbox to minimize potential low-level confounds in our study (Willenbockel et al., 2010). Examples of the resulting stimulus images are shown in Figure 1.

**Figure 2:**
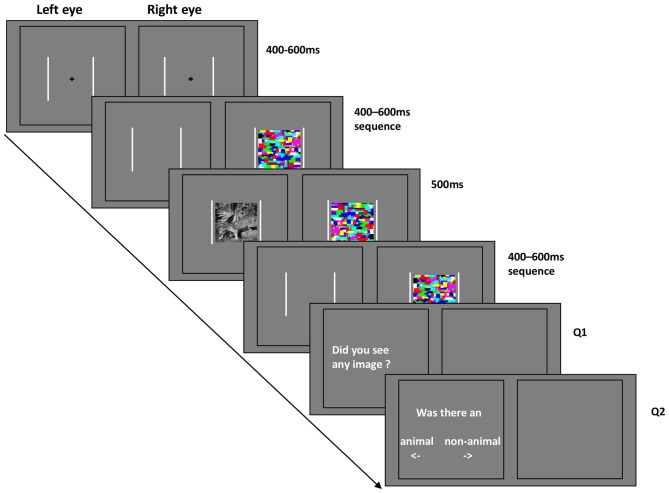
**Schematic representation of the continuous flash suppression (CFS) paradigm in Experiment 1 and 2**.

Stimuli were displayed against a gray background. All of the stimulus images were cropped into square shape extending 11.9° × 11.9°. High-contrast chromatic Mondrian CFS masks were flashed to the dominant eye at a frequency of 10 Hz, while the animal or non-animal image was presented on the other eye (Jiang et al., 2007, 2009; Zhu et al., 2016). Two white frames (13.5° × 13.5°) surrounded the outer border of stimuli and masks presented on the two sides of the screen, such that one frame was visible to each eye (see Figure 2).

Every subject completed five blocks comprised of 120 trials each. In each block 50 animal images and 50 non-animal images were shown, plus an additional 20 catch trials (without target stimulus). To prevent the effects of image-specific learning, all of the images and catch trials were presented in random order, and only once for each subject.

Each trial started with a black central fixation cross (1.2°×1.2°) shown for a random period between 400 and 600 ms (in steps of 10 ms). Subsequently, the CFS masking sequence began. After a further randomized interval of 400–600 ms, the stimuli were displayed for 500 ms with fixed contrast (without ramp). At the end of each trial, subjects were asked to answer two questions sequentially. Q1: Did you see any image? Q2: Was there an animal or non-animal? The location of the question on the screen was always the same, however the position of the words “animal” and “non-animal” were randomly switched on a trial-by-trial basis. Subjects were instructed to respond “Yes” to the appearance of any part of the stimulus images for question 1.

**Figure 3:**
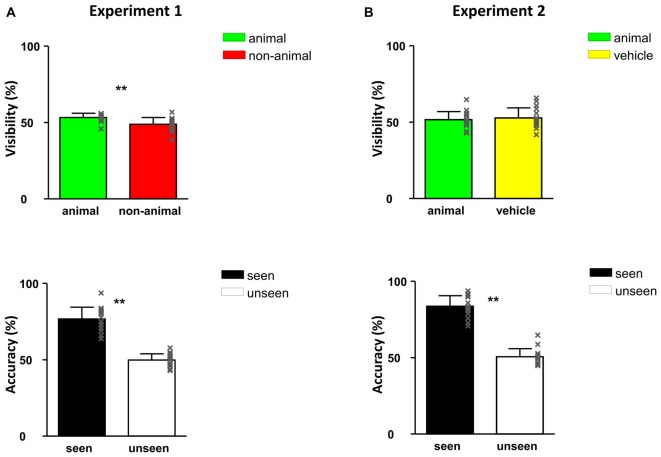
**Responses of Experiment 1 (A) and Experiment 2 (B): visibility (top) and accuracy (bottom).** All error bars represent 1 SEM. The x-shaped markers represent individual subjects. Asterisk markers designate statistical significance: *p < 0.05, **p < 0.01.

The contrast of the stimulus images was adapted by QUEST based on the response to question 1, such that the number of the images seen was maintained at around 50% of the stimuli, see Figure 3. To avoid large contrast fluctuations, the initial contrast of the QUEST procedure was adjusted for each individual subject by means of a short pretest before the formal experiments.

If subjects saw the image (“YES” in Q1), they were instructed to answer question two based on what they perceived; if they did not see the image (“NO” in Q1), they were instructed to guess the answer in question two intuitively. We informed subjects that animal and non-animal images would be presented equally often (50% ratio) in each block.

Subjects were permitted to take a break anytime during the experiment if they so desired.

#### EEG Recording and Data Analysis

Due to the contrast regulation by QUEST, the first trials of each block were sometimes shown at significantly higher or lower contrast. To avoid contamination of the results, in each block those trials that differed by more than 3 standard deviations (SD) from the convergence threshold were excluded from the analysis (trials removed per subject: avg 16%, min 10%, max 21%).

In all experiments, the subjects were fitted with a Quick-Cap (Neuroscan-USA). EEG was recorded (using a Neuroscan system) from 64 channels, based on the international 10–20 system. All electrode sites were referenced to an additional electrode on the tip of the nose. Eye movements and blinks were monitored using electrodes placed near the outer canthus of each eye (horizontal electrooculogram, HEOG), and above and below the left eye (vertical electrooculogram, VEOG). Inter-electrode impedance levels were kept below 5 kΩ. EEG was recorded continuously throughout the experiment and was bandpassed from 0.05 Hz to 100 Hz, at a 1000 Hz sampling rate. After completing data collection, the EEG recordings were segmented into 700 ms epochs, starting from 100 ms prior to stimulus onset. In addition to the manufacturer’s default eye movement removal, epochs contaminated with artifacts in any channel (the threshold for artifact rejection was manually chosen as ±80 μV in all channels) were rejected as a whole before averaging. Finally, trials were visually inspected to identify and reject trials with any remaining artifacts. ERPs were filtered digitally prior to peak detection using a bandwidth from 0.1 Hz to 30 Hz, and baseline corrected over the interval of −100 ms to 0 ms from stimulus onset.

Trials were separated into seen and unseen according to the subjects’ response to question 1. The ERP of both seen trials and unseen trials were then analyzed separately for animal and non-animal stimulus images. For the seen trials, trials with incorrect response might have been processed by our subjects as if they were either unseen trials or seen trials of the opposite target category; due to this uncertainty, only correct-response trials were included in the analysis. The area amplitudes between 150 ms and 200 ms (component 1) and between 250 ms and 300 ms (component 2) were selected as quantitative measures. These intervals have been reported to be correlated with object recognition (Thorpe et al., 1996; Johnson and Olshausen, 2003).

### Results

#### Behavioral Results

The visibility of images (determined as the percentage of trials in which subjects chose “Yes” in question 1) was 51.1%, namely around half of the images were seen in this experiment (Figure 3). When calculating the visibility of animal and non-animal trials separately, the visibility of animal images was higher than non-animal images: 53.3% vs. 48.9% (*F*_(1,30)_ = 11.67, *p* = 0.002). This shows that at the same contrast, animal images are slightly but significantly “easier to see” than non-animal images in CFS condition.

Discrimination accuracy of seen trials reached 76.8% correct, indicating that subjects could not have been guessing and therefore really “saw” the images. On the other hand, responses were only 49.9% correct in the unseen trials (seen vs. unseen: *F*_(1,30)_ = 58.36, *p* < 0.001), confirming that the subjects were guessing about the identity because they did not see the target stimulus. This pattern indicates a conservative criterion for judging awareness: participants only responded “seen” when they were able to clearly see and categorize the image.

#### ERP Results

**Figure 4:**
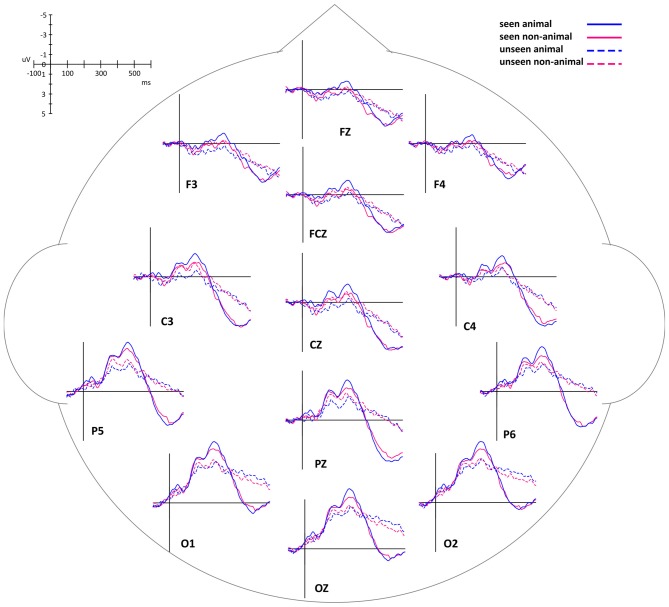
**Event-related potential (ERP) waveforms of animal and non-animal stimuli in seen and unseen trials, aligned to stimulus onset**.

By comparing average ERP waveforms of animal and non-animal stimuli on both seen and unseen trials, the activation difference between animal and non-animal images was found to be opposite between seen and unseen trials: on the seen trials, animal images induced bigger (more negative) activation than non-animal images; on the unseen trials, animal images induced smaller (less negative) activation than non-animal images (Figure 4). This overall pattern of results was then investigated using specific statistical tests, described below.

**Figure 5:**
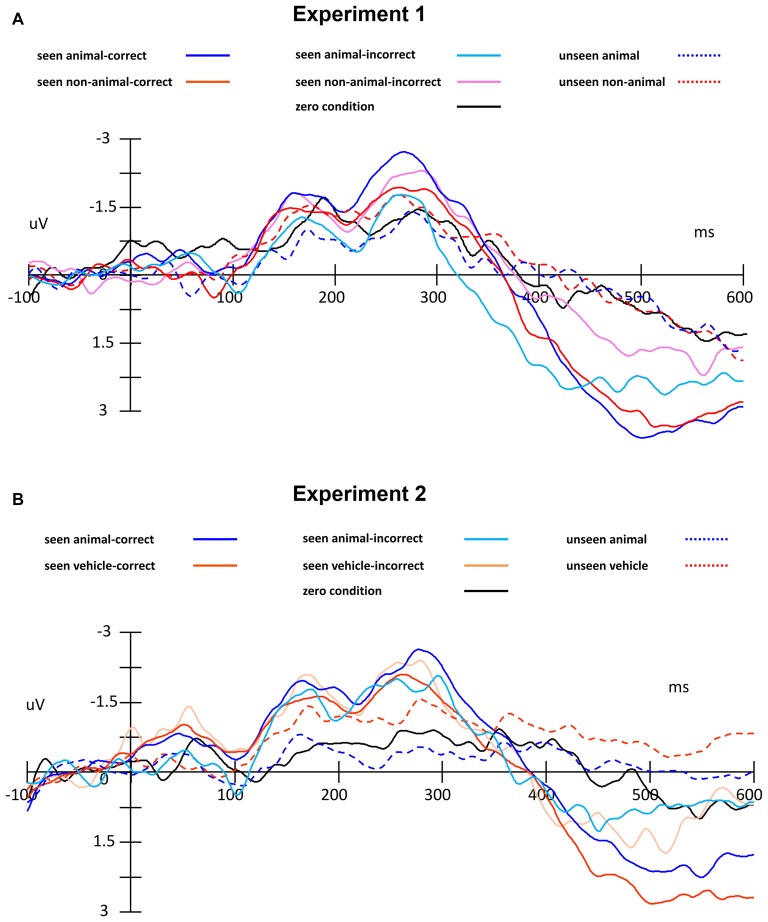
**ERP traces, separated by condition, including zero condition (catch trials without target) and error trials. (A)** Experiment 1, **(B)** Experiment 2.

**Figure 6:**
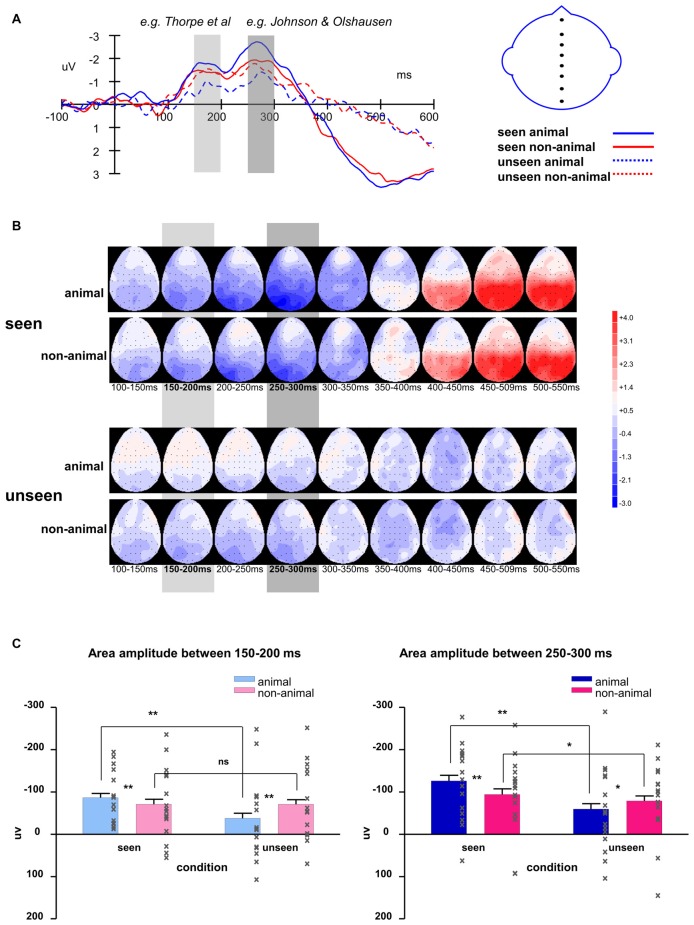
**(A)** Averaged waveforms of eight midline electrodes and **(B)** topography for animal and non-animal stimuli in seen and unseen trials; **(C)** averaged area amplitude between 150–200 ms and 250–300 ms. All error bars represent 1 SEM. Asterisk markers designate statistical significance: *p < 0.05, **p < 0.01.

In this experiment, the stimuli were always shown to the non-dominant eye and the masking sequence was shown to the dominant eye, as determined by the ABC test (Miles, 1929, 1930). In consideration of possible lateralized differences, the waveforms of the midline electrodes were analyzed first. Figure 4 shows a consistent trend of the differences between animal and non-animal images on seen and unseen trials along the midline electrodes from occipital to frontal areas. Pooled ERP waveforms of animal and non-animal stimuli on seen and unseen trials at eight midline electrodes (FPZ FZ FCZ CZ CPZ PZ POZ OZ) can be seen in Figures 5A, 6A.

The intervals of component 1 (150–200 ms) and component 2 (250–300 ms) coincide with the two most prominent negative peaks in the average waveforms (Figures 6A,B). The area amplitudes were analyzed by an ANOVA design for repeated measures with the stimulus type (animal vs. non-animal) and visibility (seen vs. unseen) as within-subjects factors (Figure 6C).

The amplitude of the seen trials was significantly bigger than those of the unseen trials for both component 1 (*F*_(1,127)_ = 31.94, *p* < 0.001) and component 2 (*F*_(1,127)_ = 41.54, *p* < 0.001). The interaction of visibility and stimulus type is significant (component 1: *F*_(1,127)_ = 31.8, *p* < 0.001; component 2: *F*_(1,127)_ = 12.8, *p* < 0.001). On the seen trials, animal images induced significantly bigger amplitudes than non-animal images (component 1: *F*_(1,127)_ = 6.84, *p* = 0.01; component 2: *F*_(1,127)_ = 20.2, *p* < 0.001); on the unseen trials, animal images induced significantly smaller amplitudes than non-animal images (component 1: *F*_(1,127)_ = 21.86, *p* < 0.001; component 2: *F*_(1,127)_ = 4.45, *p* = 0.037).

Of the 16 subjects tested in this experiment, 10 were right eye-dominant (stimuli presented to the left eye) and six were left eye-dominant (stimuli presented to the right eye). The lateral EEG electrodes of the right-dominant subjects were flipped across the midline in order to preserve possible lateralization effects. After flipping, all subjects were treated as left-dominant. Subsequently, the area amplitudes of left and right electrodes were analyzed in the same manner as the midline electrodes, as described above, except for including hemispheres (left vs. right) as the third factor. This yielded a repeated measure ANOVA with the stimulus type (animal vs. non-animal), visibility (seen vs. unseen) and hemispheres (left vs. right) as within-subjects factors. With this analysis, the activations of lateral electrodes showed a similar time course as found above with the midline electrodes. Seen trials induced bigger amplitude than unseen trials (component 1: *F*_(1,399)_ = 172.5, *p* < 0.001; component 2: *F*_(1,399)_ = 209.5, *p* < 0.001). On the seen trials, animal images induced bigger amplitudes than non-animal images (component 1: *F*_(1,127)_ = 6.84, *p* = 0.01; component 2: *F*_(1,127)_ = 20.2, *p* < 0.001); on the unseen trials, animal images induced smaller amplitudes than non-animal images (component 1: *F*_(1,127)_ = 21.86, *p* < 0.001; component 2: *F*_(1,127)_ = 4.45, *p* = 0.037). The interaction of visibility and stimulus type was significant (component 1: *F*_(1,127)_ = 44.91, *p* < 0.001; component 2: *F*_(1,127)_ = 36.76, *p* < 0.001).

Overall, the activations in the left hemisphere were larger than in the right hemisphere (component 1: *F*_(1,399)_ = 24.29, *p* < 0.001; component 2: *F*_(1,399)_ = 19.78, *p* < 0.001). For left side electrodes, animal images induced bigger amplitudes than non-animal images on the seen trials (component 1: *F*_(1,127)_ = 6.84, *p* = 0.01; component 2: *F*_(1,127)_ = 20.2, *p* < 0.001), but smaller amplitudes than non-animal images on the unseen trials (component 1: *F*_(1,127)_ = 21.86, *p* < 0.001; component 2: *F*_(1,127)_ = 4.45, *p* = 0.037). On the right brain areas, the same relations existed, but only significant for the seen trials (the interaction of visibility * stimulus type * hemisphere is significant; component 1: *F*_(1,399)_ = 17.74, *p* < 0.001; component 2: *F*_(1,127)_ = 48.35, *p* < 0.001).

Compared to component 1, component 2 had significantly larger amplitude (*F*_(1,127)_ = 16.37, *p* < 0.001). The difference between animal and non-animal/vehicle was larger for component 2 in both seen and unseen conditions (the interaction of component * stimulus type is significant; seen condition: *F*_(1,127)_ = 11.17, *p* = 0.001; unseen condition: *F*_(1,127)_ = 4.19, *p* = 0.043).

#### Summary

In this experiment, we separated trials into seen and unseen according to participant responses to question 1. From the averaged ERP waveforms and topographies (Figures 6A,B), we can see the seen trials and unseen trials were different after 350 ms (difference in area amplitude between 350–400 ms: *F*_(1,127)_ = 16.64, *p* < 0.001). While we cannot exclude that this difference was caused by response preparation or even by eye movements, it still shows that generally, seen and unseen trials were substantially different even after our main analysis intervals (components 1 and 2). This further confirmed that our design to separate seen trials and unseen trials worked as expected.

Experiment 1 showed that in the CFS conditions, the activation difference between animal and non-animal images was opposite between seen and unseen trials. In the above analysis, we treated all subjects as left-dominant by flipping the electrodes across the midline, which means that the stimuli were shown on the (normalized) right eye. We did find the activation of the left electrodes to be bigger than the right electrodes. Also, on the right hemisphere, when subjects saw the images there were different activation patterns between animal and non-animal stimuli; when subjects did not see the images, these differences were not significant.

One possible confound in our study is that animal and non-animal trials were always also object-present and object-absent trials. In animal trials, there was always a central object present (the animal). In non-animal trials, frequently the images consist of otherwise “empty” scenes (mountain, valleys, etc.), without a salient foreground object. Also, when subjects found an animal (target), they would no longer need to continue analyzing the information of the image; on the other hand, in the absence of a target, subjects may need to keep searching for a prolonged time until they can be sufficiently certain that there is no target to be found. Therefore, the type of information processing for animal and non-animal stimuli may not be fully equivalent.

Is the difference we found in Experiment 1 caused by animal detection or general object detection? Are these results “animal-specific”? In order to resolve these questions, a second object category (“vehicles”) was used to replace the unspecific “non-animals” in Experiment 2.

## Experiment 2

We compared animal and vehicle stimuli in both seen and unseen conditions by recording ERPs during a variant of the CFS paradigm.

### Methods

#### Subjects

A new set of 16 subjects participated in Experiment 2 (8 male, 8 female, aged 21–27: mean = 24.2, SD = 1.94).

#### Stimuli and Procedure

The 300 animal images were the same as in Experiment 1. In addition, 300 vehicle images were selected from the COREL database. We processed the vehicle images by the same procedure as the animal and non-animal images in order to equalize contrast and luminance across the two stimulus sets. The CFS masks were the same as in Experiment 1 as well.

The procedure of Experiment 2 was the same as in Experiment 1, except that the second question was changed into: “Was there an animal or a vehicle?”.

#### EEG Recording and Data Analysis

The EEG recording and data analysis were the same as in Experiment 1. On average, 10% (Min: 7%, Max 14%) of trials per subject were excluded from the analysis due to contrast variations resulting from the QUEST procedure.

### Results

#### Behavioral Results

In Experiment 2, as expected, around half of the images were seen (visibility: 52.2%). Importantly, the visibility of animal and vehicle stimuli was very similar, 51.6% vs. 52.8% (no significant difference, *p* > 0.05). This finding indicates that at the same contrast, animal images were not easier to detect than vehicle images in CFS conditions. As in Experiment 1, subjects had higher than chance proportion correct (83.7%) on the seen trials and around chance correct proportion (50.6%) on the unseen trials (seen vs. unseen: *F*_(1,30)_ = 23.11, *p* < 0.001). Again, this confirms that participants were accurate in their reports of seen vs. unseen stimuli.

#### ERP Results

**Figure 7:**
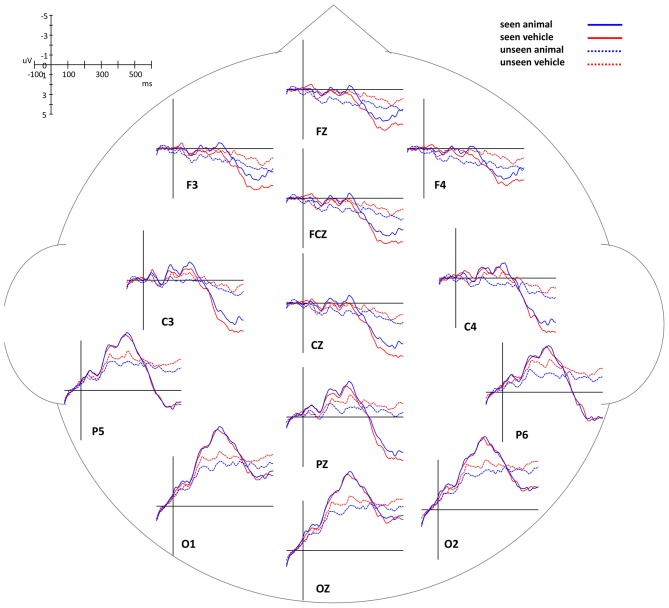
**ERP waveforms of animal and vehicle stimuli in seen and unseen trials, aligned to stimulus onset**.

**Figure 8:**
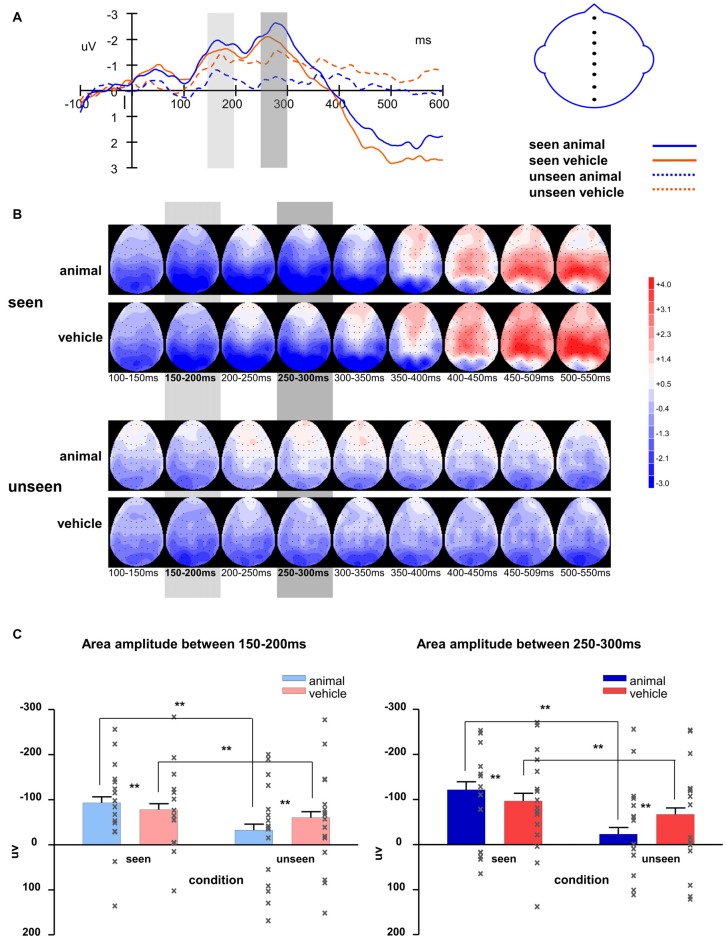
**(A)** Averaged waveforms of eight midline electrodes and **(B)** topography for animal and vehicle stimuli in seen and unseen trials; **(C)** averaged area amplitude between 150–200 ms and 250–300 ms. All error bars represent 1 SEM. Asterisk markers designate statistical significance: *p < 0.05, **p < 0.01.

The differences of the ERP waveforms between animal and vehicle images in seen and unseen trials showed trends very similar to Experiment 1 (Figures 5B, 7, 8A,B).

The waveforms of the midline electrodes were analyzed first, as seen in Figure 8C. The amplitudes of the seen trials were bigger than those of the unseen trials for both component 1 (*F*_(1,127)_ = 60.37, *p* < 0.001) and component 2 (*F*_(1,127)_ = 76.82, *p* < 0.001). The interaction of visibility and stimulus type was significant (component 1: *F*_(1,127)_ = 15.99, *p* < 0.001; component 2: *F*_(1,127)_ = 33.92, *p* < 0.001). On the seen trials, animal images induced significantly bigger amplitudes than vehicle images (component 1: *F*_(1,127)_ = 8.16, *p* = 0.005; component 2: *F*_(1,127)_ = 14.2, *p* < 0.001); on the unseen trials, animal images induced significantly smaller amplitudes than vehicle images (component 1: *F*_(1,127)_ = 11.61, *p* = 0.001; component 2: *F*_(1,127)_ = 39.2, *p* < 0.001). Compared to component 1, component 2 had larger amplitude (marginally significant, *F*_(1,127)_ = 3.12, *p* = 0.080). Similar to Experiment 1, the difference between animal and non-animal/vehicle was larger for component 2 in both seen and unseen conditions (the interaction of component * stimulus type is significant; seen condition: *F*_(1,127)_ = 5.10, *p* = 0.026; unseen condition: *F*_(1,127)_ = 9.62, *p* = 0.002).

Similar to what was found in the midline electrodes, in the lateral electrodes, seen trials induced bigger amplitude than unseen trials (component 1: *F*_(1,399)_ = 234.2, *p* < 0.001; component 2: *F*_(1,399)_ = 388.5, *p* < 0.001). Animal images induced bigger amplitudes than vehicle images on the seen trials (component 1: *F*_(1,127)_ = 19.19, *p* < 0.001; component 2: *F*_(1,127)_ = 32.97, *p* < 0.001), but smaller amplitudes on the unseen trials (component 1: *F*_(1,127)_ = 54.56, *p* < 0.001; component 2: *F*_(1,127)_ = 157.4, *p* = 0.037). The interaction of visibility and stimulus type was significant (component 1: *F*_(1,127)_ = 67.62, *p* < 0.001; component 2: *F*_(1,127)_ = 115.1, *p* < 0.001).

The activations in the left hemisphere were bigger than in the right hemisphere (component 1: *F*_(1,399)_ = 46.07, *p* < 0.001; component 2: *F*_(1,399)_ = 70.66, *p* < 0.001). Unlike the results from Experiment 1, the interaction of visibility * stimulus type * hemisphere was not significant here (component 1: *p* = 0.078; component 2: *p* = 0.25).

As in Experiment 1, the seen trials and unseen trials differed significantly after about 350 ms (difference in area amplitude between 350–400 ms: *F*_(1,127)_ = 7.24, *p* < 0.001).

#### Source Localization

**Figure 9:**
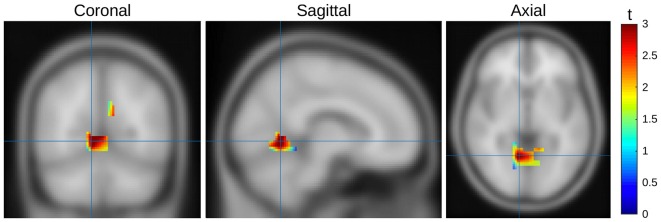
**Source mapping results, seen vs. unseen trials of both experiments, first time window.** Activity represented as *t* scores. Orthogonal slices aligned to the highest *t*-score (MNI [8 −62 4]).

In order to better understand the difference between seen and unseen trials, we estimated the location of the source. Data from Experiment 1 and 2 were pooled to minimize noise influence. To project the ERP effect into source space we used Fieldtrip (Oostenveld et al., 2011) to apply a linear constrained minimum variance beamformer approach (Van Veen et al., 1997). We used a 3-layer BEM volume conductance model as the forward model and a 1 cm spaced MNI grid (1457 points) as the lead field. Using a covariance window ranging from 100 ms to 300 ms ms after stimulus onset we first calculated a common filter for both condition types and then used these filters on the conditions separately. The spatial filters (i.e., the weight of each individual electrode to a specific source location) were calculated on both conditions together. These filters were then used for the projection of the two conditions. This two stage approach allows a better estimate of the spatial weights as more data are used to estimate the weights, and additionally the difference between conditions would be based on power differences, and not differences in spatial distribution. After calculating the source models, a dependent-samples *t*-test with the cluster based non-parametric Monte-Carlo correction was performed. As we averaged across time for the intervals windows (i.e., early 150–200 ms and late 250 ms–300 ms), cluster selection was based on spatial neighborhood. On “seen” trials during the first interval there was a significant increase in amplitude in the extrastriate cortex (Brodmann Area 19, *p* = 0.023), with a focus contralateral to the target stimulus display; there were no significant differences in the second interval (Figure 9).

#### Summary

Similar to Experiment 1, the results of Experiment 2 showed that in CFS conditions the activation difference between animal and vehicle (non-animal) images was opposite between seen and unseen trials. For seen trials, there was a larger response for animals than vehicles, while for unseen trials, the response was larger for vehicles. As in the first experiment, there was a strong modulation of visibility on the animal stimuli, but no difference between seen and unseen trials for the non-animal (vehicle) stimuli.

## Stimulus Analysis

In our experiments, the luminance and individual contrast of the images were equated (Willenbockel et al., 2010). During the experiments, the global contrast of the stimulus images was adapted continuously by QUEST to ensure around half of the images to be seen and the other half not to be seen. In order to estimate the possible effects of trial-by-trial contrast variations induced by the QUEST, the average contrast based on the subjects’ responses was computed after the experiments. The visibility was at 51.1% in Experiment 1 and 52.2% in Experiment 2, indicating that QUEST did control the number of the images seen by subjects as expected. The average contrast of seen images was only slightly higher (1.1% in Experiment 1, 2.0% in Experiment 2) than that of unseen images in both experiments. This small difference of contrast between visible and invisible images certainly minimized the possible effects of stimulus contrast on ERP results. In previous studies (Jiang and He, 2006; Jiang et al., 2009; Kaunitz et al., 2011b), high contrast images were used on “visible” trials and low contrast images were used on “invisible” trials. We used continuously adapted contrast in our experiments to avoid this imbalance between seen and unseen conditions.

The difference in contrast between seen animals and seen non-animals/vehicles, as well as the difference between unseen animals and unseen non-animals/vehicles, was negligible (0.03% and 0.26% absolute for Experiment 1, 0.30% and 0.12% absolute for Experiment 2). Likewise, the general difference of average contrast between animals and non-animals/vehicles was tiny. For details please see Table 1.

**Table 1:** **Average contrast values based on subjects’ responses**.

Experiment 1	Seen	Unseen	All	Experiment 2	Seen	Unseen	All
Animal	0.1508	0.1410	0.1462	Animal	0.1488	0.1306	0.1401
Non-animal	0.1511	0.1384	0.1454	Vehicle	0.1458	0.1318	0.1397
All	0.1510	0.1399		All	0.1471	0.1270	

**Figure 10:**
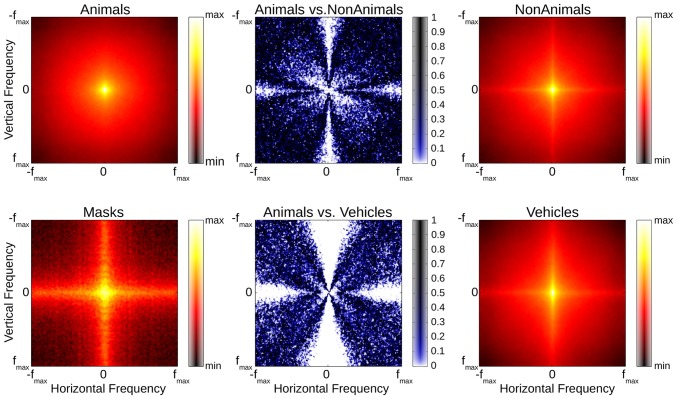
**Red/Black plots: averaged amplitude spectra of animal, non-animal, vehicle and mask stimuli (common scale).** Blue/White plots illustrate differences between stimulus classes (Kolmogorov-Smirnov-tests, computed separately per orientation and frequency pairing).

As global contrast and luminance differences cannot account for the ERP results found in our experiments, other image statistics should be considered. In order to estimate the impact a difference in the spatial frequency content of our stimulus images may have had on our ERP results, the average amplitude spectra of the stimuli images were calculated separately for each image (Wichmann et al., 2010; Zhu et al., 2013). Even though the mean of the difference between the average animal and both average non-animal and vehicle amplitude spectra was small (22% and 16% of 1 SD), Kolmogorov-Smirnov tests revealed that significant differences between animals, non-animal and vehicle stimuli were still present, particularly along the cardinal orientations (see Figure 10); these differences are largely consistent with previous studies, in which it has also been shown that amplitude spectra are not critical to human classification performance (Wichmann et al., 2010), although influences on performance are possible (Gaspar and Rousselet, 2009).

**Figure 11:**
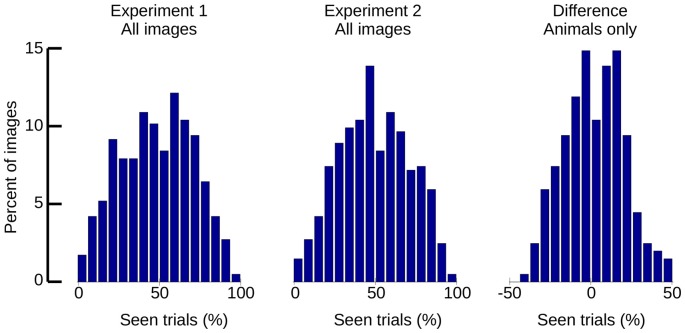
**Histograms of “seen” trials, per image.** Left: all stimuli of Experiment 1. Middle: all stimuli of Experiment 2. Right: difference between Experiments 1 and 2 (animal stimuli only).

Aside from global statistics, localized features, such as local contrast, can survive histogram normalization and have not only been shown to modulate ERPs (Groen et al., 2012, 2016), but also to be predictive of CFS performance (Moors et al., 2016). It would therefore be possible that residual features that are not necessarily related to the image category are affecting the visibility of our stimuli. We computed visibility statistics for each of our stimulus images as a general means to assess the homogeneity of our stimuli (see Figure 11). If a large number of stimuli had been seen with above or below average probability, a double-tailed distribution of visibility might be expected; if all images were detected with an even chance on every trial, a normal/Gaussian distribution centered on 50% would result. While the overall distribution of visibility per stimulus followed a Gaussian shape, Kolmogorov-Smirnov tests revealed the distributions to deviate significantly from normal (*p* < 0.001 for both experiments). Nonetheless, there was no visible degree of double-tailedness, and the majority of the images (Experiment 1: 69.3%, Experiment 2: 71.1%) fell into the central range from 25% to 75% seen. While the possibility of a small number of outliers cannot be excluded, the histograms indicate that most of our images were seen with approximately 50% chance on each trial.

Thus, it does not seem likely that the differences in the ERP signal between animal and non-animal/vehicle stimuli (and in particular the inversion between seen and unseen conditions) were trivially induced by low-level confounds.

## Discussion

### Overview

Given the high temporal resolution of neural events, ERPs are useful to reveal the time course of the neural processing underlying categorization (Thorpe et al., 1996). In our current study, ERPs were recorded during CFS to investigate the neural processes related to rapid discrimination between animal and non-animal/vehicle stimuli in the absence/presence of conscious awareness. Consistent with previous studies, we focused on two ERP components (component 1: around 150–200 ms, component 2: around 250–300 ms) that have been reported to be related to rapid categorization (Thorpe et al., 1996; Johnson and Olshausen, 2003).

### Main Results

The same pattern of results was found in both experiments. In Experiment 1, in the invisible condition, the ERPs of animal stimuli were (significantly) less negative than those of non-animal stimuli in both 150–200 ms (component 1) and 250–300 ms (component 2) after stimulus onset, which is consistent with the results in previous reports in which the targets were visible (Thorpe et al., 1996; Johnson and Olshausen, 2003). Based on this similarity between our unseen condition and the classic (seen) results, we might suspect that suppressed images are processed to a level where image content (animal or not) affects ERP waveforms. This gives rise to the hypothesis that key precursors of animal detection can take place even in the absence of explicit awareness. Under CFS conditions, although the continuously flashed masks effectively suppress the activation in the ventral pathway, some information related to suppressed stimuli might still arrive at higher brain areas (Jiang and He, 2006; Zhang et al., 2009; Meng et al., 2012). Alternatively, some information related to discriminating animal and non-animal stimuli may reach higher brain areas via a subcortical pathway that bypasses the cortical site of interocular suppression (Pasley et al., 2004). In either case, the pattern of results as discovered by Thorpe et al. (1996) may persist, at attenuated amplitude, in the unseen trials in our study.

### Result Interpretation

This original (as in Thorpe et al., 1996) pattern of results may be explained by assuming that the overall energy expended on examining the content of a stimulus image is large compared to the specific amount of energy associated with the actual detection of an animal target. In this case, the neural signature of the generic examination process might be expected to dominate the normal ERP signal. On scenes with an animal present, this examination process may not be required to be completed—the examination process may finish after the detection of the animal target. However, on “empty” scenes (without an animal target), the detection would be required to finish completely, leading to a relatively larger expenditure of energy and in consequence a stronger ERP signature. This would be true for generic non-animal scenes (without target object), but also for Vehicle scenes (with target object, but no animal): if our subjects really looked for the animal only (for if there was no animal, then it must have been a vehicle, Drewes et al., 2015), then the emerging pattern may be expected to indeed be the same.

Alternatively, when searching for an animal target, the visual brain may emphasize certain sets of feature detectors relevant for the sought-out target category. Participants may have been expecting an animal image, for example, leading to reduced activity when this prediction was confirmed rather than violated (Stefanics et al., 2014). If these detectors returned a mismatch signal at all those image locations with no relevant features present, than the sum mismatch signal on no-animal scenes may be larger than on animal scenes. Such a feature-detection process would be expected to happen relatively early in the visual processing stream and might therefore be located before the site of suppression under CFS (Crouzet et al., 2012).

In the unseen condition, the resulting ERP signature resulting from early visual areas might survive, even though further processing along the ventral pathway is effectively denied by the CFS suppression. In our seen condition, however, animal images induced bigger (more negative) activation than non-animal images, which differs from previous results (Thorpe et al., 1996). This may be explained if in addition to the previous pattern, we assume the existence of an ERP signature resultant from the specific event of a detected animal target reaching awareness. While the amplitude of this signal may be small compared to the generic ERP signature as found by Thorpe et al. (1996), it may—in the seen case—still be more robust against the consequences of CFS. While the generally larger ERP signature of the classic (non-CFS) results was effectively attenuated by the CFS masking, this specific detection signal may survive the CFS interference and hence be detected in the ERPs of our seen animal trials, explaining the inverse pattern compared to the unseen condition. A possible explanation for such signal-specific robustness might be a special neural pathway that at least partially bypasses the locus of interocular suppression (Pasley et al., 2004). One potential candidate is the amygdala, given prior evidence for a selective response to animal images (Mormann et al., 2011), as well as studies showing amygdala responses to unseen stimuli (Morris et al., 1999; Freeman et al., 2014; Troiani et al., 2014). Privileged processing of animal stimuli in itself has been postulated in the past, with the most prominent motivation for the existence of such special treatment being evolutionary necessity (New et al., 2007; Ohman, 2007; Mahon et al., 2009; Mormann et al., 2011; Crouzet et al., 2012; Yang et al., 2012; Drewes et al., 2015). Alternatively, the locus of origin of this signal might already be beyond the site of suppression.

One potential explanation for at least some findings showing an influence of unseen stimuli is that CFS may allow for the initial processing of the target stimulus but then the flashed Mondrian mask interrupts this process prior to completion (Zhu et al., 2016). If so, then visual features that are processed more quickly could activate feature/category-specific neurons prior to disruption from masking. Consistent with this idea, detection performance can improve for longer presentation durations, even under CFS, up to a plateau at around 100–150 ms (Kaunitz et al., 2014). If “animal detectors”, either in the cortex or amygdala, are activated within that time frame, then it might yield a selective response prior to the new CFS mask interrupting visual processing. Recurrent/feedback processing has been argued to play a critical role in a stimulus reaching awareness (Fahrenfort et al., 2007; van Loon et al., 2012; Koivisto et al., 2014; Moors et al., 2016). The repeated masks in the CFS paradigm might disrupt this feedback process. In the current study, the Mondrians were flashed every 100 ms, whereas recurrent/feedback processes may take 100–200 ms or more (for review: Tapia and Beck, 2014). Consistent with this idea, Koivisto et al. (2014) have argued that recurrent processing plays a key role in seeing an image (awareness) but may be less important for rapid categorization.

### Comparison With Previous Studies

The results from Experiment 1 might be affected by the possibly unequal information processing between animal and non-animal stimuli, as animal and non-animal trials are also mostly object-present and object-absent trials. In order to control for this possibility, in Experiment 2 vehicle stimuli replaced the non-animal stimuli so that there was always an object present. We found that the ERP traces were very similar as in Experiment 1, thus we can exclude the interpretation that the different activations we found between animal and non-animal stimuli might be the difference between object-present and object-absent trials.

We found the effects in component 1 and 2 to be highly similar. In the unseen condition, waveforms showed strongly reduced amplitude, and the difference between animal and non-animal ERPs was relatively uniform across and between both components. In the seen condition, the difference between animal and non-animal stimuli was larger during the second component. Thorpe et al. (1996) found significant differences between animal and non-animal stimuli as well as no-go specific activity as early as 150 ms (component 1), suggesting possible completion of visual target examination as early as component 1. Johnson and Olshausen (2003) however found that only the latter component (component 2) was significantly related to subject performance, and concluded that the first component was related to early visual processing, while only the second component was related to object recognition. Our results demonstrated significantly differing ERPs between target categories in component 1, which were further enhanced in component 2 only in the seen condition. Our data appears to be compatible with the findings of Thorpe et al. (1996), with the differences in the seen condition caused by the effects of the CFS masking. The increase in separation between seen animals and non-animals during component 2 may be interpreted as an indication that further processing has occurred, possibly relevant to object recognition as suggested by Johnson and Olshausen (2003).

### Source Localization

For a detailed source localization further data collection with higher-density recording techniques, or 3D imaging methods would be required; still, our source projection of the EEG signal related to differences between seen and unseen trials revealed higher activity in seen trials in extrastriate cortex (BA19), which is involved in a multitude of visual functionality including feature-based attention (Kamitani and Tong, 2006) and detection of patterns (Rossion et al., 2003). It is also part of both ventral and dorsal pathways and would therefore be expected to be activated in most object detection/discrimination tasks, which again would likely happen in a more pronounced way in seen trials. Thus, we conclude that the main results presented above are unlikely to be affected by unintentional confounds and do indeed reflect the differences between consciously seen and unseen visual processing of our stimuli.

### Stimulus Visibility

In the current study, the visibility of the stimulus images was measured by means of the subjects’ responses during the experiments. In previous studies, for the invisible condition, masks and stimuli were displayed to subjects and subsequently the trials when subjects saw the stimuli were discarded. On the other hand, during the visible condition, instead of masking, the stimulus image was presented to both eyes (Jiang et al., 2009). Here, we presented masks and stimuli identically in both visible and invisible trials, creating a much better balance between conditions. Approximately, half of the images were reported to have been seen and the other half was reported not to have been seen, which provided evidence that the masks and the controlling of the stimulus contrast worked as expected. The discrimination accuracies (Experiment 1: 76.8%; Experiment 2: 83.7%) were higher than chance level on the seen trials and at chance on unseen trials. This confirmed that the suppressed images were truly invisible in the unseen trials. Therefore, visibility and accuracy provided objective measures of the suppression effectiveness in our experiments.

If our findings indicate privileged processing of animal images, why did this not result in an asymmetry of the number of seen animal and non-animal/vehicle images? Perhaps the most important design aspect of this study is the constant adjustment of the overall contrast to maintain approximately 50% visibility. This was done by adjusting the main stimulus contrast, with one identical contrast for both animal and non-animal stimuli. When presented, stimuli did not fade in slowly as in a break-through paradigm; instead, they were presented suddenly at the titrated contrast level. Outside of CFS, this sudden onset would be expected to create a fairly strong visual event, while the rest of the 500 ms stimulus presentation time would have been comparatively unremarkable. In our CFS paradigm, the question whether any given trial would result in a “seen” stimulus may therefore have depended on the first few milliseconds of stimulus presentation—based on the sudden stimulus onset, rather than stimulus content. After successful stimulus detection however, the visual system would have had about 500 ms to interpret the content of the stimulus, allowing for a high hit ratio. Whether any one stimulus managed to fully penetrate into conscious perception or remained unseen may then have depended on multiple factors such as the internal state of the brain (VanRullen et al., 2011), or a possibly very minute difference in masking power of the currently displayed CFS mask. The question of whether conscious awareness is gradual or dichotomous is still a matter of controversy (Sergent and Dehaene, 2004; Overgaard et al., 2006). Many recent studies have used more gradual measures, using scales or similar methods (Overgaard et al., 2006). While such an approach could be interesting in the future to further understand the pattern of results found here, our main aim was to produce an equal number of “seen” and “unseen” trials across subjects and to ensure that targets were not detected in “unseen” trials. While the nature of the instructions given to our subjects would ensure that the “unseen” stimuli were truly invisible, the “seen” stimuli may have contained a certain proportion of stimuli that were in fact only partially “seen”. Indeed, the measured accuracy for seen trials was not perfect (76.8%, 83.7%).

### Summary

In summary, we found opposite ERP differences of animal and non-animal/vehicle stimuli between seen and unseen conditions: animal images induced bigger (more negative) activation than non-animal/vehicle images on the seen trials, yet smaller (less negative) activation on the unseen trials. These findings are consistent with arguments for a privileged kind of processing of animal stimuli, even under suppression.

## Author Contributions

JD and WZ designed the research. WZ performed data collection. JD, WZ and NAP analyzed the data. JD, WZ, NAP and DM wrote the manuscript.

## Conflict of Interest Statement

The authors declare that the research was conducted in the absence of any commercial or financial relationships that could be construed as a potential conflict of interest.
